# Paroxetine Administration Affects Microbiota and Bile Acid Levels in Mice

**DOI:** 10.3389/fpsyt.2020.00518

**Published:** 2020-06-04

**Authors:** Frederik Dethloff, Fernando Vargas, Emmanuel Elijah, Robert Quinn, Dong Ik Park, David P. Herzog, Marianne B. Müller, Emily C. Gentry, Rob Knight, Antonio Gonzalez, Pieter C. Dorrestein, Christoph W. Turck

**Affiliations:** ^1^Department of Translational Research in Psychiatry, Max Planck Institute of Psychiatry, Munich, Germany; ^2^Skaggs School of Pharmacy and Pharmaceutical Sciences, University of California, San Diego, La Jolla, CA , United States; ^3^Collaborative Mass Spectrometry Innovation Center, Skaggs School of Pharmacy and Pharmaceutical Sciences, University of California, San Diego, La Jolla, CA, United States; ^4^Division of Biological Science, University of California, San Diego, La Jolla, CA, United States; ^5^Laboratory of Translational Psychiatry, Department of Psychiatry and Psychotherapy & Focus Program Translational Neuroscience, Johannes Gutenberg University Medical Center, Mainz, Germany; ^6^Department of Pediatrics, Bioengineering and Computer Science and Engineering, and Center for Microbiome Innovation, University of California, San Diego, La Jolla, CA, United States; ^7^Department of Pediatrics, University of California, San Diego, La Jolla, CA, United States

**Keywords:** antidepressant, paroxetine, metabolomics, bile acids, microbiome

## Abstract

Recent interest in the role of microbiota in health and disease has implicated gut microbiota dysbiosis in psychiatric disorders including major depressive disorder. Several antidepressant drugs that belong to the class of selective serotonin reuptake inhibitors have been found to display antimicrobial activities. In fact, one of the first antidepressants discovered serendipitously in the 1950s, the monoamine-oxidase inhibitor Iproniazid, was a drug used for the treatment of tuberculosis. In the current study we chronically treated DBA/2J mice for 2 weeks with paroxetine, a selective serotonin reuptake inhibitor, and collected fecal pellets as a proxy for the gut microbiota from the animals after 7 and 14 days. Behavioral testing with the forced swim test revealed significant differences between paroxetine- and vehicle-treated mice. Untargeted mass spectrometry and 16S rRNA profiling of fecal pellet extracts showed several primary and secondary bile acid level, and microbiota alpha diversity differences, respectively between paroxetine- and vehicle-treated mice, suggesting that microbiota functions are altered by the drug. In addition to their lipid absorbing activities bile acids have important signaling activities and have been associated with gastrointestinal diseases and colorectal cancer. Antidepressant drugs like paroxetine should therefore be used with caution to prevent undesirable side effects.

## Introduction

Selective serotonin reuptake inhibitors (SSRIs) are commonly used drugs for the treatment of depression, post-traumatic stress disorder, generalized anxiety disorder, and other psychiatric disorders. However, a favorable patient response to SSRIs is not guaranteed, and currently, there are no biomarkers that can predict a positive treatment response, thereby preventing their strategic utilization to treat psychiatric disorders (1). Furthermore, SSRI treatment can have side effects such as diarrhea, headaches, insomnia, nausea, and weight gain, preventing it from being routinely used (2). A more thorough investigation into the mechanistic behavior of SSRI drugs is critical for optimizing drug efficacy to improve patient outcomes and minimize side effects.

The gut-brain axis (GBA) is a bidirectional mode of communication between the central nervous system (CNS) and the enteric nervous system which uses the vagus nerve to coordinate the nervous system, the endocrine system, and the immune system. Recent studies have shown that the GBA is mediated by the production of microbially secreted molecular constituents that impact CNS function and behavior relevant to psychiatric disorders (3–9). Because SSRI drugs are administered orally, it is reasonable to assume that they can alter the gut microbiota and their functions, thereby impacting other body organs including the brain (10). In this regard it has been shown that medications can affect gut physiology by altering the intestinal microbiota which can result in unfavorable side effects that include constipation and tissue toxicity (11)

In mice, bile acids are synthesized from cholesterol and after conjugation to taurine are secreted into the bile and then to the small intestine. In the intestine bile acids are deconjugated by microbes and further modified by different reactions resulting in a variety of different secondary bile acids, whose numbers are still growing. Most of the bile acids are reabsorbed in the ileum and recycled with only a small amount (~2%) found in the colon and feces (12).

Bile acids are critical for lipid digestion and absorption and drug absorption, impacting drug pharmacokinetics (13). In addition, bile acids represent signaling molecules for several nuclear receptors with functions in lipid and glucose metabolism gene expression (14). Secondary bile acids, such as deoxycholic acid and lithocholic acid, produced by the gut microbiota have functions in host metabolic processes, drug metabolism, and immune response (15–17). Depending on the drug dose and length of treatment this could be the cause for some of the observed side effects associated with SSRI treatment.

In order to assess the chronic effects of SSRI treatment, we examined fecal pellets from DBA/2J mice that were treated for 2 weeks with paroxetine (PARO). Each fecal pellet extract was then subjected to 16S rRNA sequencing and liquid chromatography tandem mass spectrometry (LC-MS/MS) analyses to delineate bacterial taxa and metabolite profiles, respectively, that differ between PARO- and vehicle (VEH)-treated mice.

## Methods

### Animal Housing and Husbandry

The experiments were carried out with male DBA/2J mice (Charles River Laboratories, Chatillon-sur-Chalaronne, France) with ages ranging between 8 and 10 weeks old. Prior to the beginning of the experiments, all animals were single-housed for at least 1 week. The mice were held under normal light and temperature conditions (12-h light/dark light cycle, lights on at 7:00 pm, temperature at 22 ± 2°C, and humidity at 55 ± 5%) with standard bedding and nesting material, in polycarbonate cages (21 cm × 15 cm × 14 cm). Water and standard housing food pellets (ssniff-Spezialdiäten GmbH, Soest, Germany) were provided *ad libitum*. All procedures were carried out in accordance with the European Communities Council Directive 2010/63/EU and approved by the local animal welfare authority (G 15-1-041, Landesuntersuchungsamt Rheinland-Pfalz, Koblenz, Germany).

### Drug Administration

To mimic clinical conditions as close as possible, animals received customized palatable pills (18), with a concentration of 5 mg/kg body weight paroxetine hydrochloride (Sigma Aldrich, Germany) or vehicle. We randomized the mice to treatment or control groups. Three days before the start of treatment, all mice were habituated to voluntary intake of vehicle pills given twice daily. Subsequently, mice received PARO or VEH pills twice daily. Compliance for each animal at the time of every drug administration (twice daily) was closely monitored by screening the cages for remnants of pills. The custom-made mouse pills were colored and can be easily recognized in the mouse cage in case the animal did not eat it immediately. Mice that did not eat two or more pills per day over the 14 days treatment schedule were excluded from analyses. This resulted in 17 animals each for the PARO and VEH groups.

### Behavioral Analysis

Forced Swim Test (FST). We introduced 2 weeks PARO- (n = 10) and VEH-treated (n = 10) mice into a 2-L glass beaker (diameter 13 cm, height 24 cm) filled with tap water (21 ± 1°C) to a height of 15 cm. We videotaped the mice for 5 min and floating and active coping behavior was scored by an experienced, treatment-blinded observer.

### Stool Collection and DNA and Metabolite Extraction

Fecal pellets were collected from the same mice following 7 and 14 days of PARO- or VEH-treatment 2 hours prior to behavioral analysis, snap-frozen on dry ice, and stored at −80°C prior to 16S and metabolomics extraction. Fecal samples were dried *via* a centrifugal low-pressure SpeedVac Plus system (Savant, Hyannis, MA) and their dry weights in microcentrifuge tubes recorded. 150 µl of sterile DEPC water and a chemically cleaned stainless-steel bead were added aseptically to each sample and followed by tissue homogenization using a TissueLyzer II (Qiagen, Hilden, Germany) for 5 min at 25 Hz. Samples were centrifuged at 33,000*g* for 10 min and the resulting supernatant aspirated into a DNA/RNA free tube and stored at −80°C for 16S sequence analysis. One milliliter 50% methanol was added to the remaining fecal homogenate and sonicated again for 5 min at 25 Hz. Following centrifugation at 33,000*g* for 10 min, the supernatant was aspirated into a clean microcentrifuge tube, dried in a SpeedVac, and stored at −80°C until mass spectrometry analysis. Samples were resuspended with 150 µl 50:50 methanol/water spiked with 2 µM sulfadimethoxine and 2 µM sulfamethazine prior to mass spectrometry analysis.

### 16S rRNA and Bioinformatic Analyses

Samples were processed following the Earth Microbiome Project 16S protocol. In short, DNA from samples was extracted *via* the MoBio PowerSoil kit, then extracts were amplified using PCR (515F/806R primers: 5′-GTGCCAGCMGCCGCGGTAA-3′ and 5′-GGACTACHVGGGTWTCTAAT-3′, respectively), which targets the V4 region of the 16S ribosomal subunit. Amplicons were sequenced using the Illumina MiSeq platform at the “Genomic Center of the University of California, San Diego.” Metadata and microbiome data were uploaded and processed using the Qiita software (19). Raw sequences were demultiplexed and quality controlled as defined in the default processing within Qiita, and the resulting sequences were collapsed into Amplicon Sequence Variants (ASV) using Deblur 1.1.0. Further analyses were performed using QIIME2 as implemented in Qiita (19). For this purpose, the ASV table was rarefied at 5,000 sequences per sample. Rarefaction is the process of randomly subsampling without replacing the number of sequences within a sample, where we discard any sample below the given threshold. This helps with possible biases while characterizing the beta diversity measurements. Then, to compare the microbial community structure of the treatment and control groups, we used phylogenetic metrics of alpha (within-sample) and beta-diversity (between samples). Specifically, we used Faith's Phylogenetic Diversity for alpha diversity, and the weighted and unweighted UniFrac distances for beta diversity (20). Sample sizes were n=8 for paroxetine 1 week group, n=12 for paroxetine 2 week group, and n=20 for vehicle group. Principle coordinates analysis plots were used to visualize the beta-diversity among the samples, and PERMANOVA testing was used to identify statistically different clusters.

In addition, ASV were used for rank correlation analysis with FST and PARO and PARO metabolites (number of animals = 9). Correlation analysis of 16S ASV with behavior and drug metabolites in PARO-treated mouse feces is based on a rank correlation (Spearman). After filtering for at least five reads a list of 93 features (16S ASV) was used to perform correlation analysis. Correlation coefficient cutoff was set to 0.6 for positive correlations and −0.6 for negative correlations. The correlated ASV were then summarized at phylum level (**Table 1**).

**Table 1:** Number of Bacteria in a phylum correlate with behavior and drug treatment.

	Bacteroidetes	Firmicutes	Others
**neg corr to FST**	10	1	
**Pos corr to PARO**	8	2	
**Pos corr to PARO M I**	6	3	1
**Pos corr to PARO M III**		4	1
**Pos Corr to FST**		4	
**Neg corr to PARO**		6	
**Neg corr to PARO M I**		6	1
**Neg corr to PARO M III**		7	1

Number of animals = 9, number of ASV = 93 after filtering for 60% data coverage.

Negative correlations (NEG CORR), r ≤ −0.6; positive correlations (POS CORR), r ≥ 0.6); forced swim test (FST); Paroxetine (PARO); Paroxetine metabolite I (PARO M I); Paroxetine metabolite III (PARO M III).

### Metabolomics Analysis

Mouse fecal pellet extracts from PARO- (n = 17) and VEH-treated (n = 17) mice were analyzed using an ultra-high pressure liquid chromatography system (Vanquish, Thermo Scientific, Waltham, MA) coupled to a Q Exactive mass spectrometer (Thermo Scientific) fitted with a heated electrospray ionization (HESI-II, Thermo Scientific) probe. Chromatographic separation was accomplished using a Kinetex C18, 1.7 µm, 100 Å, 2.1 mm × 50 mm column fitted with a C18 guard cartridge (Phenomenex, Torrance, CA) with a flow rate of 0.5 ml/min. Five microliters of extract was injected per sample/QC. The column compartment and autosampler were held at 40°C and 4°C, respectively, throughout all runs. Mobile phase composition was: A, LC-MS grade water with 0.1% formic acid (v/v) and B, LC-MS grade acetonitrile with 0.1% formic acid (v/v). The chromatographic elution gradient was: 0.0 to 1.0 min, 5% B; 1.0 to 9.0 min, 100% B; 9.0 to 11.0 min, 100% B; 11.0 to 11.5 min, 5% B; and 11.5 to 12.5 min, 5% B. Heated electrospray ionization parameters were: spray voltage, 3.5 kV; capillary temperature, 380.0°C; sheath gas flow rate, 60.0 (arbitrary units); auxiliary gas flow rate, 20.0 (arbitrary units); auxiliary gas heater temperature, 300.0°C; and S-lens RF, 60 (arbitrary units). Mass spectrometry data was acquired in positive mode using a data dependent method with a resolution of 35,000 in MS1 and a resolution of 17,000 in MS2. An MS1 scan from 100 to 1500 m/z was followed by an MS2 scan, using collision-induced dissociation of the five most abundant ions from the prior MS1 scan.

### Metabolomics Data Pre-Processing and Statistical Analysis

The acquired mass spectrometry data was converted to the open mzXML format using MSconvert from ProteoWizard (21). Files were then further processed with the implemented ADAP-modules (22) in MZmine II (v. 2.35) (23) to generate MS1 features with associated peak area and MS2 scans (parameters are listed in **Supplementary Table 1**). The “export to GNPS” module on MZmine II was used to generate MS1 (quant.csv) and MS2 (.mgf) files for use in the feature based molecular networking workflow (https://ccms-ucsd.github.io/GNPSDocumentation/featurebasedmolecularnetworking/) on the GNPS website (http://gnps.ucsd.edu). The data was filtered by removing all MS/MS fragment ions within ± 17 Da of the precursor m/z. MS/MS spectra were window filtered by choosing only the top 6 fragment ions in the ± 50 Da window throughout the spectrum. The precursor ion mass tolerance was set to 0.02 Da and MS/MS fragment ion tolerance of 0.02 Da. A network was then created where edges were filtered to have a cosine score above 0.7 and more than six matched peaks. Edges between two nodes were kept in the network only if each of the nodes appeared in each other's respective top 10 most similar nodes. Finally, the maximum size of a molecular family was set to 100, and the lowest scoring edges were removed from molecular families until the molecular family size was below this threshold. The spectra in the network were then searched against GNPS spectral libraries. The library spectra were filtered in the same manner as the input data. All matches kept between network spectra, and library spectra were required to have a score above 0.7 and at least six matched peaks.

Metabolic features were annotated by searching the GNPS (https://gnps.ucsd.edu) network and matching MS2 spectra. After GNPS networking we searched specifically for MS2 spectra that matched to bile acids. In order to remove redundancy and increase specificity, we manually extracted single peaks of individual bile acid compounds and adducts of molecular ions when possible (**Supplementary Table 2**).

For statistical analysis the Perseus software (v1.5.5.3) (24) was used. Raw peak intensities of metabolic features were log2-transformed, missing values were estimated by replacing from normal distribution (width, 0.3; downshift, 1.8). Two sided T-test was performed. For correlation analysis of raw peak intensities and experimental meta data (“correlogram”), the corrplot package (v0.84) (https://cran.r-project.org/web/packages/corrplot/index.html) in R with RStudio (v1.1.456) (https://www.rstudio.com/) was used.

If not stated otherwise, graphs and tests were performed in Microsoft Excel.

## Results and Discussion

There are different approaches to model depression-like phenotypes (i.e. symptoms of depression) in the mouse. While induction of depression-like symptoms following exposure to different types of stress, e.g. chronic social defeat or chronic mild stress is one possible approach, the use of mouse strains with high innate anxiety- and depression-like behavior is also commonly accepted. The DBA/2J strain can be considered a “depressed” mouse strain. The selection of the DBA/2J mouse strain with its well-described high innate anxiety and responsiveness to antidepressant treatment (18) enabled us to perform the pharmacological treatment under basal conditions, i.e. without the need to subject the animals to an additional stress procedure that might have influenced microbiome data.

In previous studies, we and others have shown that PARO-treated DBA/2J mice respond with less floating in the FST and increased time spent in the lit compartment of the dark light box, indicating lower depression-like behavior and lower anxiety, respectively (18, 25–29). In line with previous experiments, we fed mice with either VEH- or PARO-containing pills (2 × 5 mg/kg/day) for 14 days. Compared to direct injection, this method of drug administration is less stressful for the animal and mitigates the effects of confounding behavioral assays used to assess depression-like behavior. In addition, oral administration is the preferred method considering the aim of the study to analyze the effects of the drug on gut microbiota. PARO pill consumption was carefully monitored to ensure that every animal was subjected to the same amount of the drug, resulting in 17 animals for the VEH-treated group and 16 animals for the PARO-treated group.

**Figure 1:**
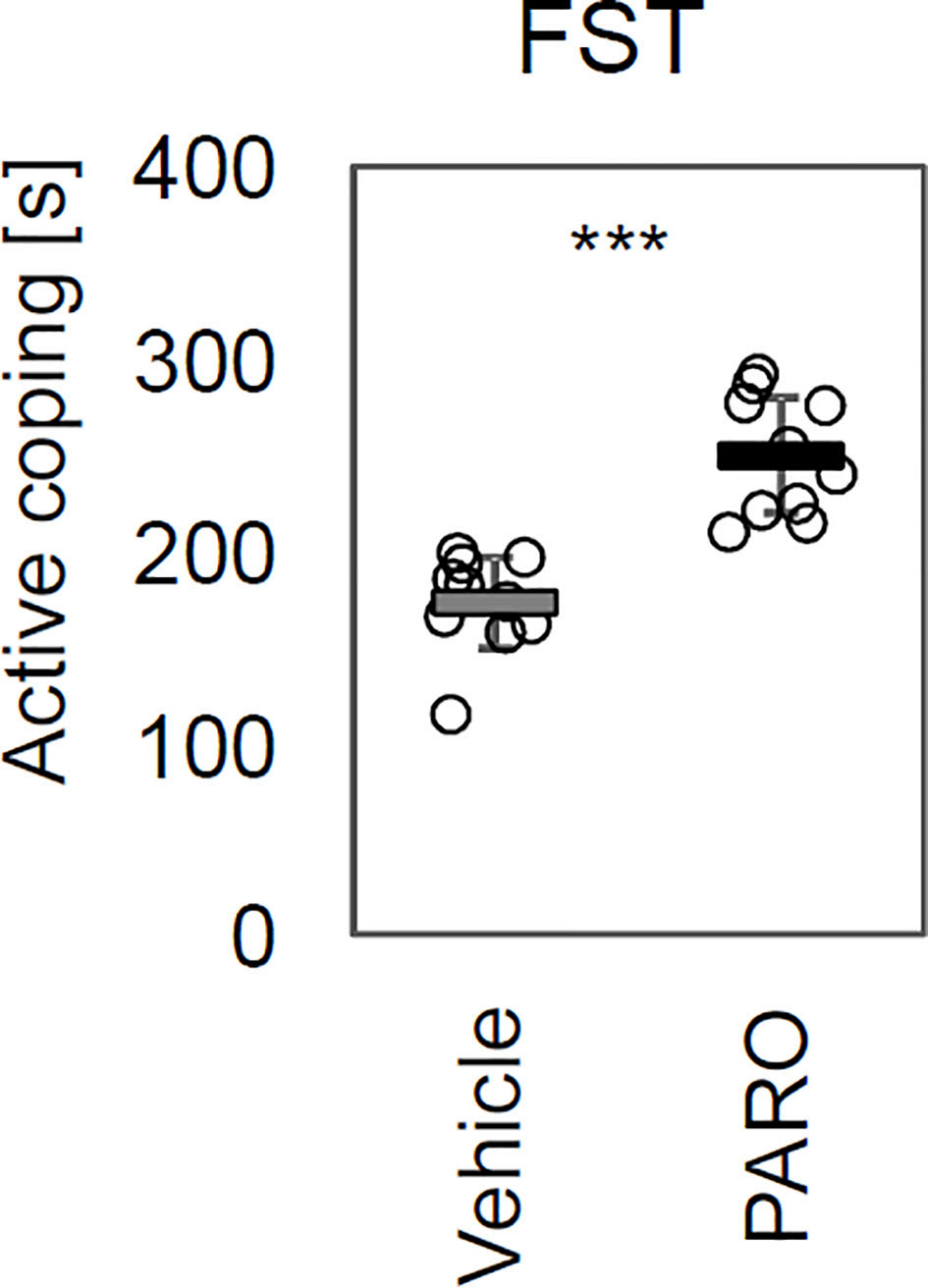
Paroxetine treatment revealed antidepressant-like and anxiolytic effects in DBA/2J mice. Chronic paroxetine (PARO) treatment for 14 days increased time of active coping (p = 1.5 E-7) in the Forced Swim Test (FST). Sample size was n = 10 for PARO group and n = 10 for Vehicle group. Bars represent mean ± SD, ***p < 0.001.

Confirming results from previous experiments (25–27, 29), mice of the PARO-treated group (n = 10) showed significantly higher active coping time (p = 1.5E-7) in the FST compared to VEH-treated animals (n = 10) indicating the antidepressant-like and anxiolytic effects of PARO in DBA/2J mice (**Figure 1**).

In order to investigate the effects of PARO on the gut microbiota we used fecal pellets from drug- and VEH-treated animals as a proxy. Fecal pellets were collected from the same mice following 1 and 2 weeks of daily treatments and used for 16S bacterial rRNA sequence analysis and extracted metabolites prepared for LC-MS/MS as outlined in Methods.

**Figure 2:**
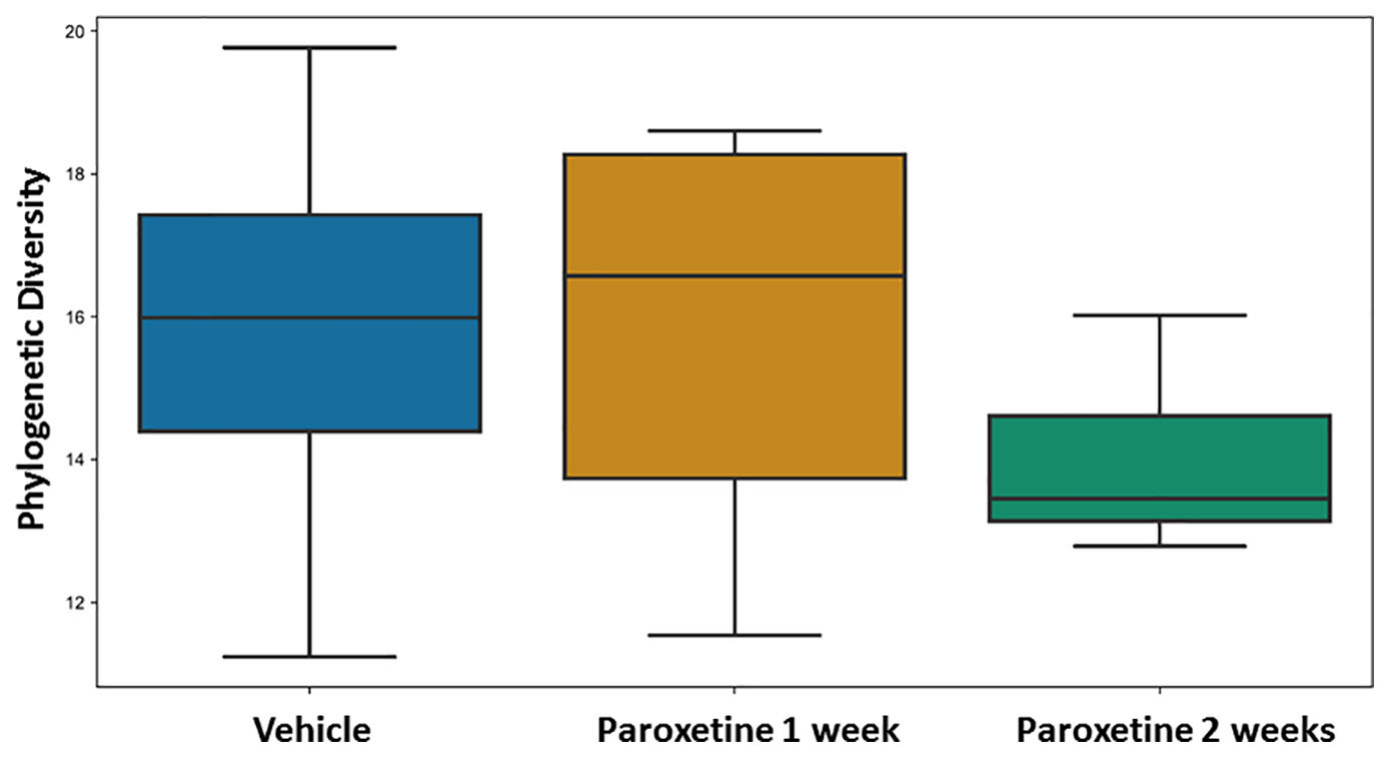
Fecal pellet alpha diversity is significantly different (p-value = 0.011402, q-value = 0.034207) in paroxetine- compared to vehicle-treated mice. Alpha-diversity was compared between treatment and control groups using Faith's Phylogenetic Diversity (PMID: 19455206). Sample sizes were n=8 for Paroxetine 1 week group, n = 12 for Paroxetine 2 week group and n = 20 for Vehicle group.

No differences were found for beta-diversity of the microbiota in the fecal pellets after 7 and 14 days of PARO treatment (**Supplementary Figure 1**) with either the weighted or unweighted UniFrac distances. However, alpha diversity showed a reduced and significant (p < 0.04) Faith's phylogenetic diversity in the 14 days PARO-treatment group (**Figure 2**). Correlation analysis based on ASV revealed that bacteria belonging to the Firmicutes phylum correlated positive with the active coping time in the FST and negative with PARO levels, while more bacteria belonging to the Bacteroidetes phylum correlated negative with the active coping time in the FST and positive with PARO levels (**Table 1**). This indicates that PARO treatment has an influence on the composition of gut microbes which may be linked with behavior.

**Figure 3:**
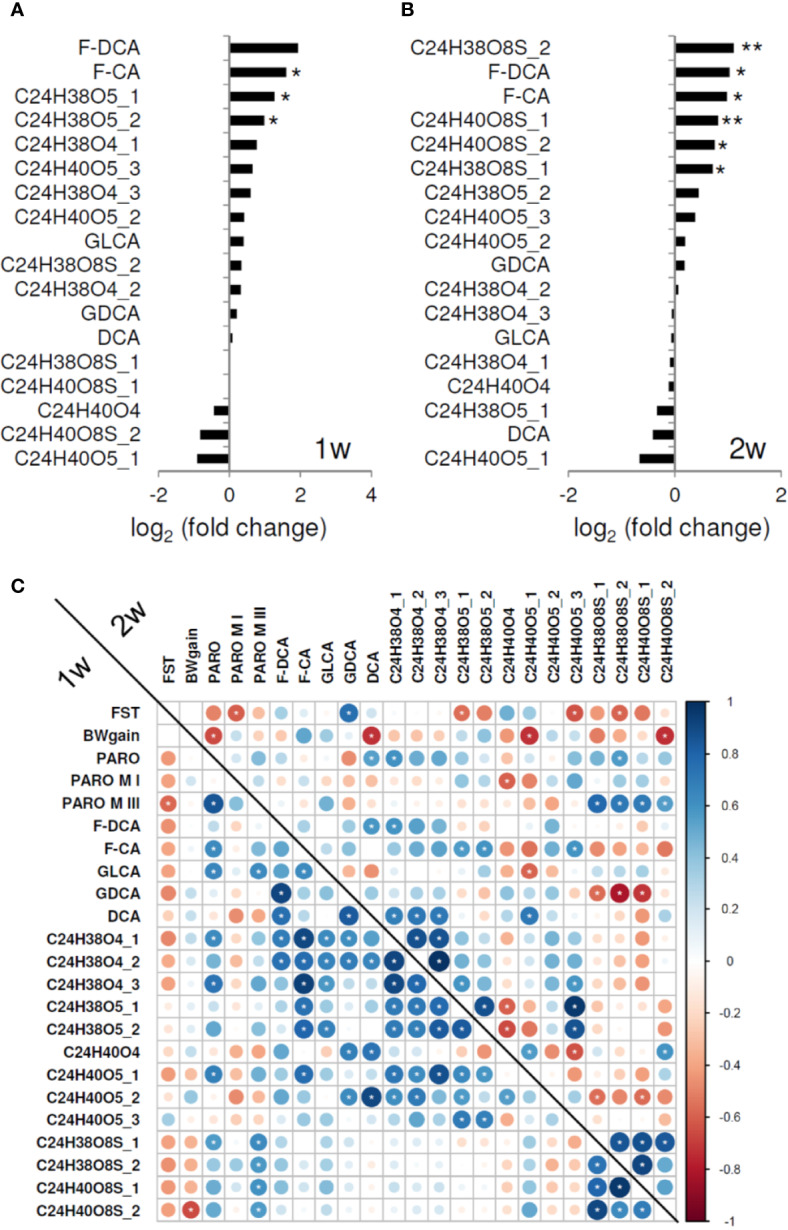
**(A)** Fecal pellet bile acid level ratios following 1 week (1w) and **(B)** 2 weeks of paroxetine (PARO) treatment, sample size n = 10 each. Bars represent mean log2 fold changes PARO *Vehicle-1. Significance was tested using Student's *t*-test; significance is indicated (p < 0.05 = *; p < 0.01= **). Several fecal pellet bile acid levels are increased following PARO treatment. **(C)** Several fecal bile acid levels correlate significantly with behavior, body weight gain, PARO metabolites, and other bile acid levels (indicated with white asterisk, p < 0.1). Correlogram displays the Pearson correlation coefficient values of treatment with PARO. Lower left represents the correlations after 1 week (1w) of treatment und upper right the correlations after 2 weeks (2w) of treatment. Size and color intensity reflect the absolute value as indicated by the color bar. Abbreviations in order of appearance: BWgain, body weight gain; FST, forced swim test; paroxetine, PARO; paroxetine metabolite I, PARO M I; paroxetine metabolite III, PARO M III; F-DCA, phenylalanodeoxycholic acid; F-CA, phenylalanocholic acid; GLCA, glycolithocholic acid; GDCA, glycodeoxycholic acid; DCA, deoxycholic acid.

Furthermore, untargeted mass spectrometry analyses revealed increased levels for 13 (3 p < 0.05) and 11 (4 p < 0.05, 2 p < 0.01) primary and secondary bile acids after 1 and 2 weeks of treatment, respectively (**Figures 3A, B**). Four sulfated bile acids with sum formulas C_24_H_38_O_8_S and C_24_H_40_O_8_S, each detected with different chromatographic retention times, showed higher levels in PARO-treated mice.

Correlation analysis (**Figure 3C**; **Supplementary Figure 2**) exposed negative correlations between body weight gain and PARO and three bile acid levels, i.e., deoxycholic acid, C_24_H_40_O_5__1 and C_24_H_40_O_8_S_2, following 2 weeks of treatment. FST active coping correlated negatively with PARO metabolites after 1 week and 2 weeks of treatment, and with several bile acid levels (C_24_H_38_O_5__1, C_24_H_40_O_5__3, C_24_H_38_O_8_S_2) after 1 week. In addition, a strong correlation of FST active coping was found with glycodeoxycholic acid after 2 weeks. Interestingly, the four sulfated bile acids negatively correlated with FST behavior and PARO metabolite III levels (p < 0.1) (**Figure 3C**). Bile acid sulfation is a known mechanism for elimination and detoxification (30, 31) by increasing compound solubility and decreasing intestinal absorption which results in higher fecal content. Greater sulfated bile acid production may thus be caused by PARO, reflecting a mechanism to dispose of high drug levels from the organism.

Of particular interest are the correlations we found with behavioral measures of the recently characterized secondary bile acids phenylalanocholic acid and phenylalanodeoxycholic acid (32) implicating that microbiota are affected in PARO-treated mice (**Figures 3A, B**; **Supplementary Figure 3**). Strong positive correlations were found for PARO and for several bile acids after 1 week. After 2 weeks phenylalanocholic acid showed a weak negative correlation with sulfated bile acids (**Figure 3C**).

Gut microbiota alterations can have an impact on bile acid levels in response to PARO treatment. This is supported by previous studies where antibiotic treatment results in primary and secondary bile acid changes (33). In this regard several studies have shown that antidepressant drugs including sertraline, fluoxetine, and PARO have antimicrobial activities that impact Gram-positive bacteria (34). Of particular significance are recent findings that implicate bile acids in several gastrointestinal disorders (35–38) and intestinal tumorigenesis through their interaction with the nuclear farnesoid X receptor (FXR) (39, 40).

Bile acids are generated in the liver from cholesterol by a multi-step mechanism involving 17 enzymes and metabolized in the intestine by the gut microbiota (41). Subsequent deconjugation takes place in gut residing bacteria with the help of bile salt hydrolase activity. Following deconjugation a small fraction of primary bile acids that do not undergo resorption enter the colon, where they are processed into secondary bile acids by *Clostridium* and *Eubacterium* (42–45). They can be either absorbed by passive processes or excreted in the feces. Secondary bile acids synthesized by the microbiota only make up a very small fraction of the total bile acid pool. Which steps of primary and secondary bile acid synthesis are affected by PARO treatment is unknown and awaits further studies on the mechanism of action of the drug in bacteria.

Numerous reports have provided ample evidence for a link between the gut and the CNS affecting its physiology, function, and ultimately behavior (46–49). In this regard gut microbiota composition has been shown to impact behavior related to social activity, stress response, and anxiety, which are all relevant for the pathology of major depressive disorder (MDD) and other psychiatric disorders (50–52).

In order for microbiota to exert their function it is believed that microbial molecules serve as mediators that ultimately affect targets in the CNS (53–58). Most studies investigating microbiota and their molecular constituents have been carried out in animals. In one example extinction learning deficits in germ free mice was associated with altered microbiota-derived metabolite levels (51). Other work with germ-free mice has revealed reduced anxiety and altered “hypothalamic pituitary adrenal axis” function compared to control mice (59). A limited number of studies also revealed microbiota alterations in MDD patients compared to controls (3, 60).

With regard to the effects of antidepressant drugs a recent study carried out in a trait anxiety rat model on the effects of minocycline, an antibiotic that also has antidepressant activities, has found changes in microbial composition and microbial metabolites in response to drug treatment. Rats treated with minocycline had a reduced number of microglial cells in the prefrontal cortex implicating a role for microbiota composition in immune function that is mediated by the gut-brain-axis (61).

Of particular interest for our findings is a recent report that presents evidence for an involvement of drugs including antidepressants on microbiota composition (62). Since bile acids are processed by microbiota it is not at all surprising that their composition and levels are affected by dysbiosis. However, at present, it is unclear how altered bile acid compositions and levels might affect CNS physiology and behavior.

A change in bile acid composition and levels have been associated with several other diseases including intestinal dysmotility, inflammatory bowel disease phenotypes, nonalcoholic fatty liver disease, and progression of colon cancer (63, 64). Microbiota that impact secondary bile acids are an additional factor that when altered can contribute to health and disease by affecting body weight, lipid metabolism, intestinal mucosal function, and cardiovascular function (12, 65, 66).

Our results suggest that antidepressant drugs like PARO should be used with caution to prevent undesirable side effects. Whether bile acid levels can serve as biomarkers for monitoring the SSRI treatment response remains to be tested with a greater number of animals to allow for stratification of drug responders and non-responders.

## Data Availability Statement

The original contributions presented in the study are publicly available. The mass spectrometry data have been deposited to the Center for Computational Mass Spectrometry (MSV000085446). 16S rRNA data have been deposited at European Nucleotide Archive (PRJEB34564).

## Ethics Statement

The animal study was reviewed and approved by Landesuntersuchungsamt Rheinland-Pfalz, Koblenz, Germany.

## Author Contributions

FD and FV did the metabolomics processing and data and correlation analyses. EE, RQ, AG, and RK did the processing, alpha- and beta-diversity test on the microbiome. DP, DH, and MM did the mice housing and behavior tests. EG, PD, CT, and all authors contributed to writing the manuscript.

## Conflict of Interest

The authors declare that the research was conducted in the absence of any commercial or financial relationships that could be construed as a potential conflict of interest.
