# Supplementary material for: Paroxetine Administration Affects Microbiota and Bile Acid Levels in Mice
Source: Front Psychiatry. 2020 Jun 4;11:518. doi: 10.3389/fpsyt.2020.00518 (PMC7287167; doi:10.3389/fpsyt.2020.00518)
Supplement: Supplementary file 1 [file DataSheet_1.pdf]

Paroxetine administration affects microbiota and bile acid levels in mice

Supplementary Material

Frederik Dethloff[1*], Fernando Vargas[2,3,4*], Emmanuel Elijah[2,3], Robert Quinn[2,3§], Dong Ik Park[1#], David P. Herzog[5], Marianne B. Müller[5], Emily C. Gentry[2,3], Rob Knight[6], Antonio Gonzalez[7], Pieter C. Dorrestein[2,3], Christoph W. Turck[1+]

[1] Max Planck Institute of Psychiatry, Department of Translational Research in Psychiatry, Munich, Germany.
[2] Skaggs School of Pharmacy and Pharmaceutical Sciences, University of California, San Diego, La Jolla, CA, USA.
[3] Collaborative Mass Spectrometry Innovation Center, Skaggs School of Pharmacy and Pharmaceutical Sciences, University of California, San Diego, La Jolla, CA, USA.
[4] Division of Biological Science, University of California, San Diego, La Jolla, CA, USA.
[5] Laboratory of Translational Psychiatry, Department of Psychiatry and Psychotherapy & Focus Program Translational Neuroscience, Johannes Gutenberg University Medical Center, Mainz, Germany.
[6] Department of Pediatrics, Bioengineering and Computer Science and Engineering, and Center for Microbiome Innovation, University of California, San Diego, La Jolla, CA, USA
[7] Department of Pediatrics, University of California, San Diego, La Jolla, CA, USA

[*] These authors contributed equally to this work.

[§] Present address:
Michigan State University, Department of Biochemistry and Molecular Biology, 603 Wilson Rd, Rm 116B, East Lansing, MI 48824, USA

[#] Present address:
Aarhus University, Department of Biomedicine, Ole Worms Allé 3, 8000 Aarhus C, Denmark

[+] Corresponding author:

Christoph W. Turck
Max Planck Institute of Psychiatry
Kraepelinstrasse 2-10
80804 Munich, Germany

Phone: +49-89-30622317
E-mail: turck@psych.mpg.de

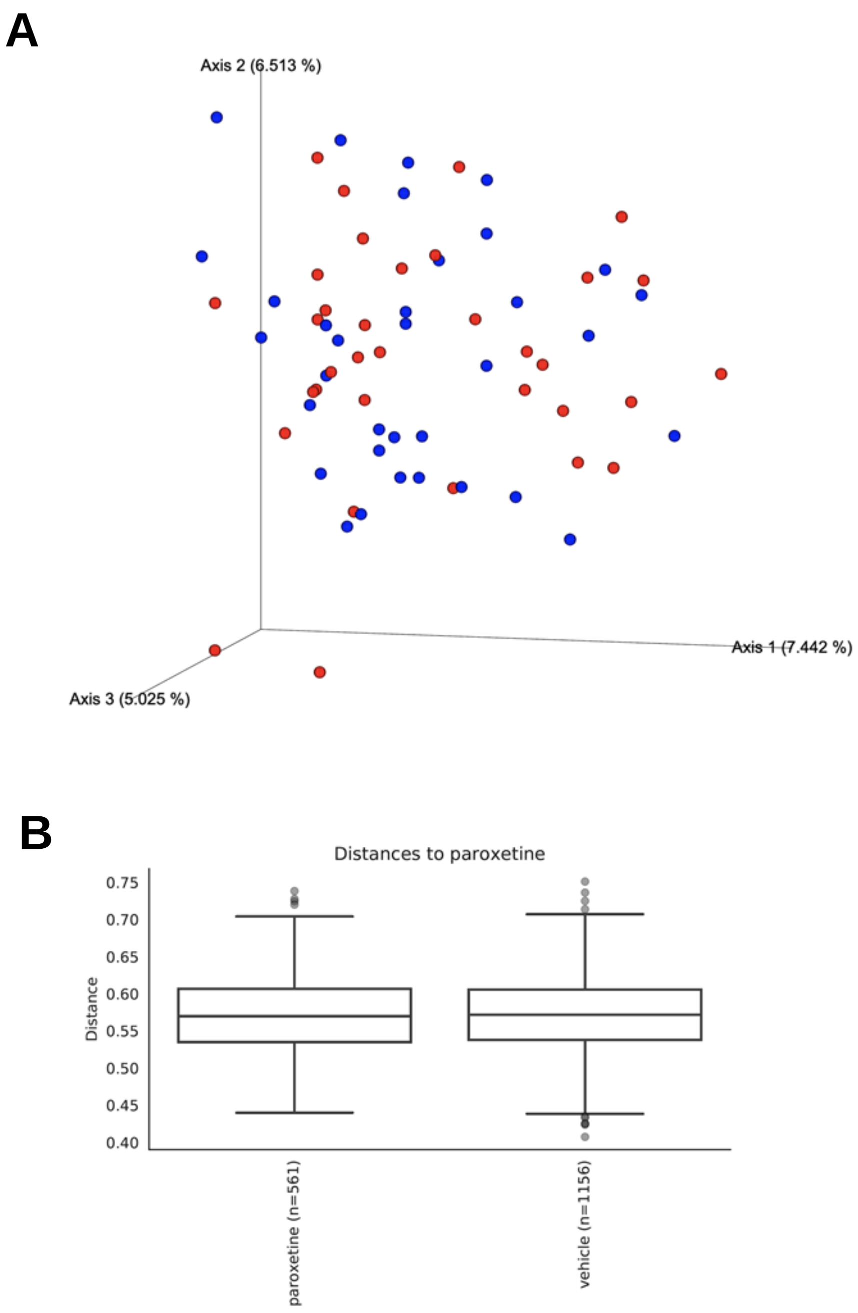

Supplementary Figure 1. (A) Unweighted UniFrac microbiome distances projected using principle coordinate analysis for analysis of beta-diversity of the microbiome data. Blue spheres are fecal samples from vehicle-treated mice (n=34), red spheres are samples from paroxetine-treated mice (n=34). (B) Boxplots of unweighted UniFrac distances of samples in PCoA plot above used to calculate PERMANOVA testing. p-value from the PERMANOVA test is 0.982.

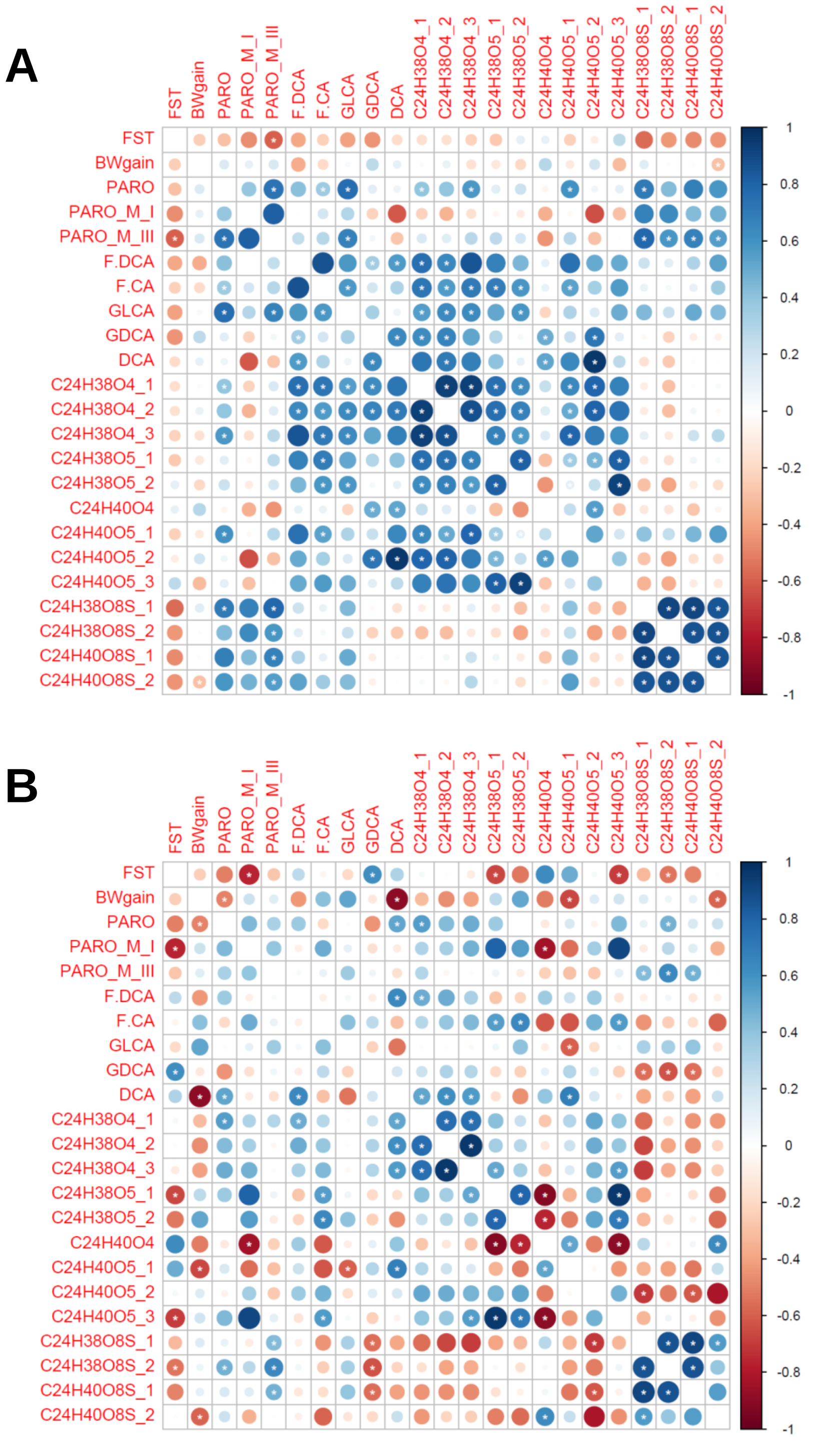

Supplementary Figure 2: Fecal pellet bile acid levels are associated with body weight gain, behavior, and other bile acid levels. Correlation matrix is based on Spearman correlation. Significance is indicated by white asterisk ($p < 0.1$). (A) One week treatment and (B) two weeks treatment. Correlations are based on the paroxetine (PARO)-treated mouse group (n=10).

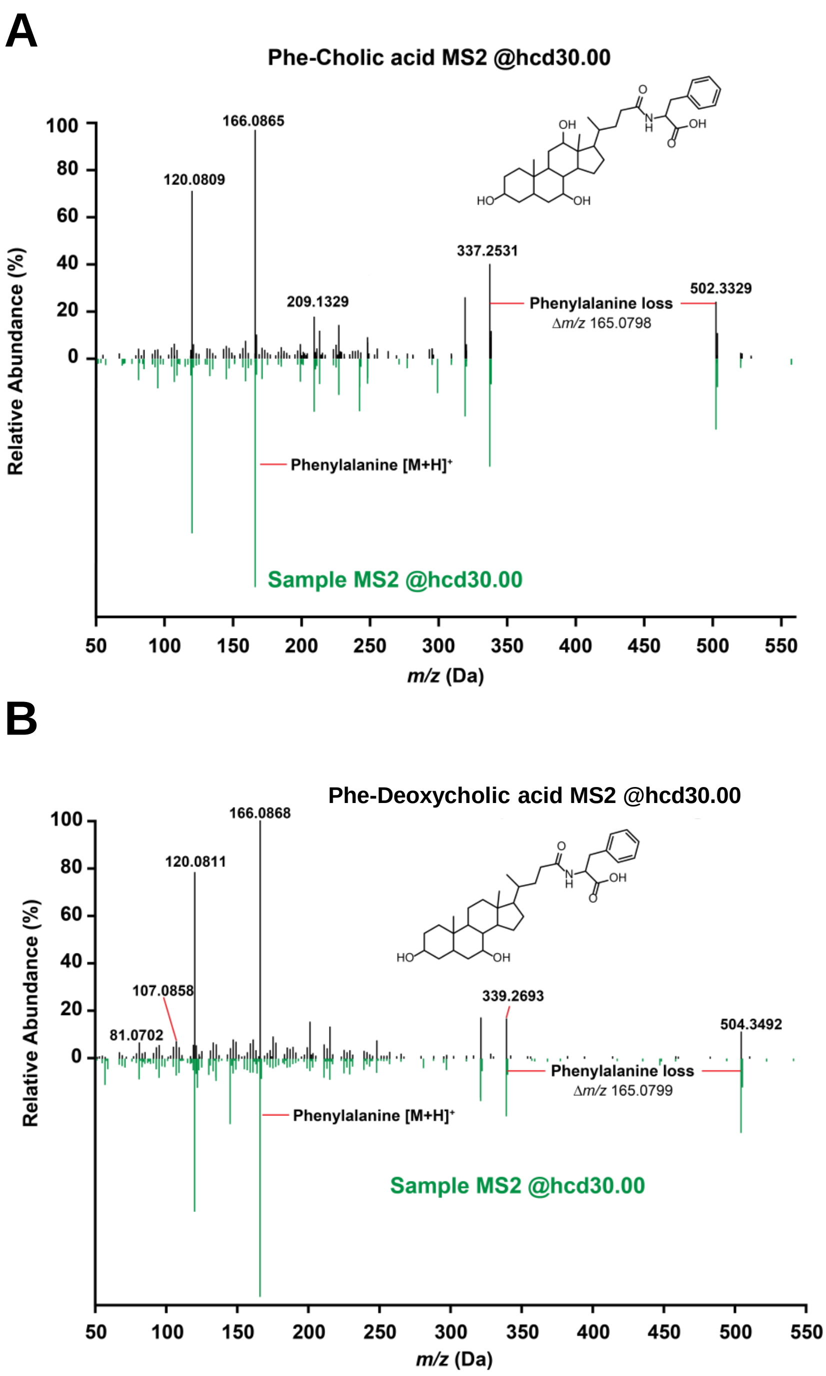

Supplementary Figure 3: (A) MS2 spectrum of the newly found phenylalanocholic acid (Phe-Cholic acid) molecule in the sample mirrored against the Phe-Cholic acid molecule found in the GNPS database. (B) MS2 spectrum of the newly found phenylalanodeoxycholic acid (Phe-Deoxycholic acid) molecule in the sample mirrored against the Phe-Deoxycholic acid molecule found in the GNPS database. Second best hit was to phenylchenodeoxycholic acid spectrum.

Supplementary Table 1: Parameters used for MZmine feature detection

Crop filtering

- Retention time: 0 – 5.6 min
- m/z: 50 – 5990

Mass detection

- MS1 mass detector: 2.0 E5
- MS2 mass detector: 1.0 E2

ADAP Chromatogram builder

- Min group size in # scans 5
- Group intensity threshold 2.0E5
- Min time span (min): 0.05
- Min highest intensity: 6.0 E5
- m/z tolerance: 0.005 m/z or 10 ppm

Chromatogram deconvolution

- Local min search
  - Chromatogram threshold: 0.01%
  - Search minimum in RT range (min): 0.40
  - Min relative height: 0.01%
  - Min absolute height: 6.0 E5
  - Min ration of peak top/edge: 3
  - Peak duration range (min): 0.05 – 0.40
- m/z range for MS2 scan pairing (Da): 0.05
- RT range for MS2 scan pairing (min): 0.2

Isotope peak grouper:

- m/z tolerance: 0.01 m/z or 10 ppm
- Retention time tolerance (min): 0.1
- Maximum charge: 4

Join Alignment

- m/z tolerance: 0.005 m/z or 10 ppm
- Weight for m/z: 90
- Retention time tolerance (min): 0.3
- Weight for RT: 10

Peak list row filter

- Minimum peaks in a row: 2
- Reset the peak number ID

Gap fill

- Intensity tolerance: 20%
- m/z tolerance: 0.01 m/z or 10 ppm
- Retention time tolerance (min): 0.2

Supplementary Table 2: Metabolite and meta data from mouse behavior experiments (Supplementary Table 2.xlsx).
